# A Narrative of Intraoperative Staple Line Leaks and Bleeds During Bariatric Surgery

**DOI:** 10.1007/s11695-016-2177-1

**Published:** 2016-04-19

**Authors:** Sudip K. Ghosh, Sanjoy Roy, Ed Chekan, Elliott J. Fegelman

**Affiliations:** Global Health Economics and Market Access, Ethicon Inc., 4545 Creek Road ML 96, Cincinnati, OH USA; Medical Affairs, Ethicon Inc., Cincinnati, OH USA

**Keywords:** Bariatric surgery, Intraoperative intervention, Laparoscopic, Sleeve gastrectomy, Gastric bypass, Gastric leak, Gastric bleed, Staple line

## Abstract

The primary objective of this review was to assess the incidence of intraoperative staple line leaks and bleeds during laparoscopic sleeve gastrectomy (LSG) and laparoscopic Roux-en-Y gastric bypass (LRYGB). A literature search of MEDLINE®, EMBASE™, and Biosis from January 2010 to November 2014, plus secondary citations extending to 2008, identified 16 relevant articles. For LSG, the incidence of intraoperative leaks and bleeds was as high as 3.93 and 4.07 %, respectively. For LRYGB, leaks occurred in up to 8.26 % and bleeds in 3.45 % of cases. Stapler misfire was commonly cited as a cause. Widespread, precautionary use of staple line reinforcement (SLR), lack of standardized testing, and underreporting suggest the incidence may be underestimated. Published studies were insufficient to address the economic impact of bleeds and leaks or interventions, but development of improved stapler designs that obviate the need for SLR may reduce costs and improve outcomes.

## Introduction

Bariatric surgery is an effective surgical option for morbid obesity that provides substantial and sustained weight loss with concomitant resolution of comorbidities [1–3]. Roux-en-Y gastric bypass surgery was one of the earliest and most effective surgical methods for managing obesity [4]. Further refinements included a transition from open to laparoscopic surgery, thereby decreasing patient discomfort and allowing faster recovery while reducing the duration of hospitalization along with other medical costs [5–7]. Sleeve gastrectomy was initially used in the super obese (BMI ≥ 50) [8] as the first step of a staged operation, to reduce weight to a safer level before undergoing a more complex surgery such as biliopancreatic diversion or gastric bypass. Because the resulting weight loss and resolution of comorbidities obtained with laparoscopic sleeve gastrectomy (LSG) were comparable to laparoscopic Roux-en-Y gastric bypass (LRYGB), LSG became an appealing alternative as a simpler, safer procedure [9]. A number of studies support the use of LSG, including the most current American Society for Metabolic and Bariatric Surgery position statement [10].

Despite evidence that laparoscopic procedures have improved morbidity and mortality, LSG and LRYGB still carry some risks [11]. Staple line integrity is critical to creating a functional anastomosis (LRYGB) or an effective sleeve (LSG) and has been the focus of continuing innovation by surgical stapler manufacturers [12]. Staple line failure with gastric leak is one of the most serious and feared complications for both LSG and LRYGB. The post-operative leak rate for LSG varies between 1 and 3 % for the primary procedure, whereas the reported incidence of leaks for LRYGB varies from 0.1 to 5.6 % [6, 10, 13, 14]. The long staple line in LSG creates a propensity for leaks, especially near the gastroesophageal junction (GEJ) [15]. These leaks are more difficult to resolve, potentially because of high gastric pressure along with acid and bile in the gastric sleeve. Staple line failure is also the most common cause of post-operative gastrointestinal (GI) hemorrhage for both LSG and LRYGB, with reported incidences of 1–3 and 1.9–4.4 %, respectively [10, 16]. Technical aspects of stapling can vary, and factors such as anatomical location, tissue viscosity, staple height, and other intrinsic properties of the stapling system itself may substantially influence staple formation [15]. Many studies acknowledge that the experience of the surgeon is critical in creating an anastomosis with sufficient staple line integrity to resist leakage and promote healing [17–19]. In a recent review, more collaboration between surgeons and device manufacturers was encouraged to reduce complications and improve patient outcomes [12].

In contrast to the predominance of data on post-operative leaks and hemorrhage, documentation of intraoperative staple line leaks and bleeds is inconsistent or absent in most published studies on LSG or LRYGB. The rate of intraoperative events appears to be relatively low but may vary depending on the surgeon’s learning curve or other ancillary methods of prevention that are used routinely such as oversewing, staple line reinforcement (SLR) using buttressing materials, and the use of tissue sealants or glues. These variables could potentially represent hidden costs, either directly or through increases in operative time, length of stay, and post-operative factors such as infections. Thus, the primary objective of this review was to assess the incidence of intraoperative staple line leaks and bleeds during the two most common procedures for bariatric surgery, LSG and LRYGB. Relevant studies were further evaluated for the use of intraoperative leak testing and staple line interventions, as well as the potential impact on complications and clinical efficiency factors.

## Methods

### Literature Search Strategy

A search of the medical literature was conducted to identify publications describing intraoperative bleeds, leaks, and interventions at the staple line for LSG and LRYGB. The databases MEDLINE®, EMBASE™, and Biosis were restricted to a search period from January 2010 to June 2015. Titles were searched using the key words laparoscopic sleeve gastrectomy *or* sleeve gastrectomy *or* gastric bypass. The results were filtered using a key word search of the full citation, abstract, and descriptor for staple line *or* laparoscopic *or* leak *or* bleed *and* intraoperative *or* intervention, with appropriate truncations. Case reports were excluded.

Intraoperative leaks were identified based on a positive intraoperative leak test, whereas intraoperative bleeds were identified based on surgeon examination of the staple line that may or may not require adjunct staple line interventions.

## Results

### Literature Search

**Fig. 1:**
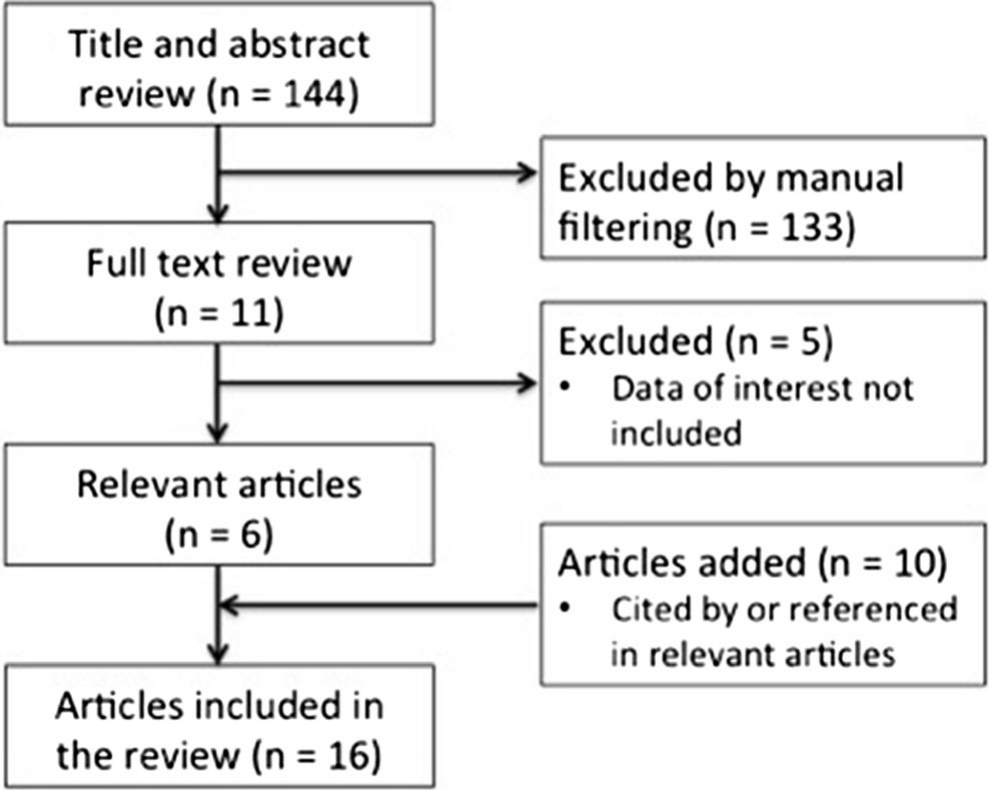
Flow diagram illustrating the selection process used for publications included in the current review

The literature search yielded a total of 144 titles and abstracts, all of which were manually filtered (Fig. 1). From these, 10 full articles that reported on results of intraoperative leak testing or intraoperative observation of staple line bleeding incidents were reviewed. Articles that did not contain relevant data, including the detection of intraoperative leaks and/or bleeds, were excluded, leaving six articles for inclusion. Ten additional references dating from January 2008 to November 2013 were identified as cited by or referenced in the filtered articles. Because the methods used to collect data were heterogeneous across the final 16 publications, this study was structured as a narrative, rather than a systematic review. Of the 12 LSG articles, nine reported clinical data directly, with one study each performed in facilities in the USA, Israel, India, Lebanon, Belgium, Italy, and Australia, and two studies conducted jointly in Kuwait and Egypt. Of the four LRYGB studies, three were performed in the USA, and one was conducted jointly in Kuwait and the USA.

### Laparoscopic Sleeve Gastrectomy

Three recent literature reviews addressed the incidence of intraoperative leak testing in LSG as well as recommendations for leak prevention and management. A systematic review by Parikh et al. [20] evaluated 112 studies encompassing 9991 patients and found that intraoperative leak testing was performed in 6717 patients (67 %) and 62 studies (55 %). Although the goal of this study was to identify surgical strategies to prevent leaks, extensive variations in stapling techniques precluded their ability to conduct a meaningful statistical analysis of stapling as a factor. Aurora et al. [16] found similar results in a systematic review of 4888 patients in 29 publications, with performance of a leak test documented in 15 studies (52 %). In a review of four published studies on leak prevention and management, Abou Rached et al. [13] recommended a number of preventive measures including gentle handling of tissues, staple line reinforcement, larger bougie size, and routine use of methylene blue (MB) intraoperative leak testing.

Intraoperative complications and the value of intraoperative leak testing were considered in three retrospective analyses of patient data. Chopra et al. [21] conducted a review of 174 consecutive LSGs. Perioperative complications occurred in 26 cases (14.94 %). Two patients experienced intraoperative bleeds (1.15 %), although the site was not specified, and neither required surgical intervention. In an analysis of 2834 patients, Sakran et al. [22] found a high level of intraoperative mishaps (as defined by the author) in 14 (31.8 %) of the 44 patients that eventually developed post-operative leaks, including two patients with bleeding from the staple line that was sealed by suturing and four cases of stapler misfire that were oversewn. Thirty-three of the 44 patients that developed post-operative leaks had been tested intraoperatively using blue dye (*n* = 25) or air (*n* = 8), but only one leak was detected, suggesting that the dye test may be of limited predictive value for post-operative leaks. This occurred in a patient that had experienced a stapler misfire. Although the leak was sutured and tested negative in a subsequent dye test, the patient leaked during the post-operative period. The authors suggest that routine intraoperative leak testing of all patients is superfluous for the prevention of post-operative leaks, and that selective testing of patients with specific types of intraoperative complications such as stapler misfire may be more appropriate. In contrast, a study of 712 patients by Wahby et al. suggested that intraoperative testing was beneficial, detecting leakage in 28 cases (3.93 %) that were repaired by oversewing [23]. Intraoperative complications noted in this study also included bleeding (*n* = 3, 0.42 %) at the upper pole of the spleen that was resolved laparoscopically and 29 patients (4.07 %) with bleeding at the staple line that was controlled by endoclips or reinforcing stitches. Interestingly, no additional leaks were detected using upper GI contrast at 24–48 h. This study supports the routine use of MB intraoperatively but suggests that the value of subsequent early post-operative upper GI series is limited and accompanied by additional disadvantages such as cost, discomfort for the patient, and risks associated with irradiation and aspiration pneumonia. Taken together, these studies are consistent with the concept that detection and repair of intraoperative leaks, presumably of mechanical origin and due to technical issues with staple line integrity, are of value, even though other factors related to ischemia may impact the development of later, post-operative leaks [24].

Additional studies of SLR provide further insight into the nature of intraoperative bleeds and leaks. In a prospective study of 75 patients by Dapri et al. [25], no reinforcement was compared with SLR using Seamguard® (W. L. Gore & Associates, Inc, Flagstaff, AZ) or suturing. Seamguard significantly reduced the volume of intraoperative blood loss compared with the other two groups, both during sectioning and overall during the procedure, whereas surgeries performed without reinforcement required significantly less time for stomach sectioning as well as total operating time. Angrisani et al. [26] enrolled 105 patients to receive LSG as a primary procedure. The staple line was buttressed using multiple blue cartridges loaded with PSD and reinforced with titanium clips while the non-buttressed transection line was reinforced using a running, absorbable sero-serosal suture. No bleeds were reported, and only one intraoperative leak occurred in a patient who received LSG 8 months after gastric band removal and had scar tissue and multiple, positive leak tests during surgery. These leaks could not be successfully repaired by suture, and the procedure was converted to a RYGB with distal gastrectomy. The four studies discussed in this and the previous paragraph suggest that SLR may provide a minor benefit in preventing intraoperative bleeds that should be balanced with other considerations including operative time and cost.

Two studies whose primary focus was safety and efficacy reported relevant information on intraoperative bleeding or leaks. In a retrospective review of 185 patients, Armstrong et al. [27] described two staple line bleeds (1.08 %) that occurred within 12 h of the operation and required reoperation via laparoscopy. In addition, one patient had a splenic bleed from an injury involving the tip of the stapler anvil, which was treated with sealant (Floseal; Baxter, Deerfield, IL). There appear to have been additional bleeds, as it is noted that staple line bleeding points were treated effectively with clips (Ligaclips®; Ethicon Endo-Surgery, Cincinnati, OH) and sprayed with fibrin glue (Tisseel; Baxter), but the actual incidence of intraoperative bleeds was not reported, and leak testing was not performed intraoperatively. Abd Ellatif et al. [28] conducted a retrospective multicenter review of 1395 subjects and identified 35 patients (2.5 %) who experienced intraoperative bleeding at the staple line, controlled by endoclips or stitching, and 28 patients (2.0 %) with intraoperative staple line leaks that were treated by oversewing. While there do not appear to be significant safety concerns regarding intraoperative leaks and bleeds during LSG, their incidence is often not reported and the costs associated with interventions have not been clearly quantified.

### Laparoscopic Roux-en-Y Gastric Bypass

Four relevant articles provided information on intraoperative bleeding and leaks during LRYGB surgery. The Longitudinal Assessment of Bariatric Surgery (LABS) study, a prospective, multicenter, longitudinal study of adverse intraoperative events and long-term outcomes that occur during and after bariatric surgery evaluated 2973 LRYGB operations during phase 1 of the study [29]. No intraoperative transfusions were necessary, although there were 40 cases (1.34 %) of organ injury requiring sutures. In 1539 patients enrolled in phase 2, there were no cases of bleeding, defined as a volume ≥2 units. The incidence of equipment failure, which included but was not restricted to stapler misfire, was 29 cases (0.98 %) in phase 1 and 12 cases (0.78 %) in phase 2. Leaks were not documented because it was determined that there was no consistent method for detection across the 10 clinical sites involved in the study.

In one of the three remaining LRYGB studies, Madan et al. [30] examined the hypothesis that intraoperative leaks at the stapled GJ site could be treated using omental reinforcement with suturing of the problem area and selective use of fibrin glue. In doing so, they found that 32 of 387 patients (8.26 %) had a staple line dehiscence or evidence of gastric pouch or GJ leak intraoperatively. To assess the role of intraoperative endoscopy in managing GJ leaks, Alasfar et al. [31] conducted a chart review of 290 patients who underwent LRYGB and had intraoperative events at the time of surgery. The anastomosis was created using a linear stapler. An intraoperative endoscopy air leak test detected leaks in 11 patients (3.79 %), with all but one located at the GJ site. Leaks were surgically corrected with oversewing and passed a subsequent leak test. Intraoperative pouch bleeding occurred in 10 cases (3.45 %). In six of these, significant bleeding from a blood vessel was visible endoscopically, and remedial suturing was performed under direct endoscopic visualization. The source could not be identified in the other four cases, but they resolved without further intervention after irrigation and evacuation of blood clots. In a study spanning from November 2001 to July 2005, Jamil et al. [32] focused on managing upper GI hemorrhage in LRGYB patients using endoscopic intervention with combination therapy including epinephrine, heater probe, and/or endoclips. A retrospective chart review was conducted in the 30 of 933 patients (3.22 %) that developed upper gastrointestinal hemorrhage (UGIH). Endoscopy in 27 of 30 patients (90 %) revealed that all had bleeding from the GJ staple line. Of the 21 (70 %) patients that developed UGIH intraoperatively or in the immediate post-operative period of less than 4 h, 5 (16.67 %) had bleeding intraoperatively and 16 (53.33 %) developed UGIH within 4 h of the operation. Blood transfusion was required in 47 % of patients with UGIH, and the mean length of stay significantly increased from 2.84 to 4.1 days. A cost analysis was not performed in these studies, but these types of complications might be expected to add to the expense of the LRYGB procedure.

## Discussion

This review of recent literature revealed that the available information on intraoperative leaks and bleeds for the two most common procedures used in bariatric surgery, LSG and LRYGB, is somewhat remarkable with regard to how infrequently and incompletely these events are currently documented and reported. A majority of publications retrieved in the original search were focused on post-operative rather than intraoperative leaks and bleeds. Although the focus of the manuscript is on intraoperative leak events, we included a few articles on post-operative leaks that referred to intraoperative leak events. Manual filtration and cross-referencing through citations were essential to uncover relevant publications, although a possible limitation is that some data embedded within articles may have been overlooked. Of the 12 LSG articles that were included, intraoperative leaks were reported in three studies, and bleeds were reported in 4 studies, with an incidence of up to 3.93 and 4.07 %, respectively. For LRYGB, two of four articles reported leaks and/or bleeds in up to 8.26 and 3.45 % of cases, respectively. While the rate of leaks appears to be higher for LRYGB, the ranges overlap. A formal comparison and potential differences related to SLR could not be conducted in this review, due to heterogeneity of methods and the small number of available studies. The etiology of intraoperative leaks is unknown and highly likely to be multifactorial, depending on patient factors, surgical technique, and device malfunction. Stapler misfires were cited as the cause of device-related staple line failures, while other studies implicated the inherent characteristics of the tissue at specific locations (such as the GEJ for LSG and the GJ site for LRYGB) as well as the experience of the surgeon [12, 15].

Two deficiencies in reporting and managing intraoperative leaks were apparent in that only 52–55 % of LSG studies employed testing [33, 20], and there is no standard testing procedure, as noted in the LABS consortium study [29]. Although some investigators have questioned its routine use, in particular as an early indicator of post-operative leaks [22], intraoperative leak testing has been used successfully to detect procedure-related leaks that occur before the patient leaves the operating room and when tissues are most amenable to repair [23]. Thus, standardization of leak testing could greatly assist in determining the incidence of leaks resulting from mechanical sources of staple line insufficiency. Similarly, methods for detecting intraoperative staple line bleeding are not standardized but present a somewhat different challenge in that bleeds are often undocumented and considered a nuisance that is treated routinely with cauterization, sutures, sealants, or clips or may self-resolve by the application of pressure along the staple line.

Few studies addressed the impact of intraoperative leaks and bleeds on other complications or efficiency factors such as operative time, cost, or length of stay. For LSG, one article indicated that while bleeds did not affect operative time, they did disrupt the momentum of the operation [34]. Only one of the four LRGYB articles assessed factors associated with UGIH such as the need for blood transfusion and increased length of stay, although an actual cost analysis was not provided [33]. A 2005 study of LRYGB calculated a significantly higher operative cost for use of SLR that averaged approximately $1600 per patient, with no differences in hospital service cost or total hospitalization cost [35]. Given that LSG and LRYGB are currently considered safe and effective bariatric options, factors that affect cost are likely to become increasingly important in future decision-making.

In conclusion, this non-systematic review of the current literature supports the premise that intraoperative staple line leaks and bleeds are primarily associated with stapler misfires. Although their incidence appears to be relatively low, it may be underestimated as a result of underreporting, the precautionary use of SLR, lack of standardized testing, and the capacity of some leaks and bleeds to resolve with little or no treatment. In the intraoperative setting, there is insufficient data to assess the economic impact of leaks and bleeds and whether the added cost of SLR can be justified by improvements in clinical efficiency or outcomes. Ultimately, development of new stapler designs that improve staple line integrity may obviate the need for SLR, increase efficiency, and lead to better outcomes for patients undergoing bariatric surgery.
